# Effects of an Overground Walking Program With a Robotic Exoskeleton on Long-Term Manual Wheelchair Users With a Chronic Spinal Cord Injury: Protocol for a Self-Controlled Interventional Study

**DOI:** 10.2196/19251

**Published:** 2020-09-24

**Authors:** Alec Bass, Mylène Aubertin-Leheudre, Claude Vincent, Antony D Karelis, Suzanne N Morin, Michelle McKerral, Cyril Duclos, Dany H Gagnon

**Affiliations:** 1 School of Rehabilitation Faculty of Medicine Université de Montréal Montréal, QC Canada; 2 Centre for Interdisciplinary Research in Rehabilitation of Greater Montreal Centre Intégré Universitaire de Santé et Services Sociaux du Centre-Sud-de-l’Île-de-Montréal Montréal, QC Canada; 3 Department of Exercise Science Université du Québec à Montréal Montréal, QC Canada; 4 Department of Rehabilitation Faculty of Medicine Université Laval Québec, QC Canada; 5 Centre for Interdisciplinary Research in Rehabilitation and Social Integration Québec, QC Canada; 6 Department of Medicine McGill University Montréal, QC Canada; 7 Departement of Psychology Faculty of Arts and Sciences Université de Montréal Montréal, QC Canada

**Keywords:** assistive technology, locomotion, spinal cord injury, rehabilitation, robotics, osteoporosis

## Abstract

**Background:**

In wheelchair users with a chronic spinal cord injury (WU_SCI_), prolonged nonactive sitting time and reduced physical activity—typically linked to this mode of mobility—contribute to the development or exacerbation of cardiorespiratory, musculoskeletal, and endocrine-metabolic health complications that are often linked to increased risks of chronic pain or psychological morbidity. Limited evidence suggests that engaging in a walking program with a wearable robotic exoskeleton may be a promising physical activity intervention to counter these detrimental health effects.

**Objective:**

This study’s overall goals are as follows: (1) to determine the effects of a 16-week wearable robotic exoskeleton–assisted walking program on organic systems, functional capacities, and multifaceted psychosocial factors and (2) to determine self-reported satisfaction and perspectives with regard to the intervention and the device.

**Methods:**

A total of 20 WU_SCI_, who have had their injuries for more than 18 months, will complete an overground wearable robotic exoskeleton–assisted walking program (34 sessions; 60 min/session) supervised by a physiotherapist over a 16-week period (one to three sessions/week). Data will be collected 1 month prior to the program, at the beginning, and at the end as well as 2 months after completing the program. Assessments will characterize sociodemographic characteristics; anthropometric parameters; sensorimotor impairments; pain; lower extremity range of motion and spasticity; wheelchair abilities; cardiorespiratory fitness; upper extremity strength; bone architecture and mineral density at the femur, tibia, and radius; total and regional body composition; health-related quality of life; and psychological health. Interviews and an online questionnaire will be conducted to measure users’ satisfaction levels and perspectives at the end of the program. Differences across measurement times will be verified using appropriate parametric or nonparametric analyses of variance for repeated measures.

**Results:**

This study is currently underway with active recruitment in Montréal, Québec, Canada. Results are expected in the spring of 2021.

**Conclusions:**

The results from this study will be essential to guide the development, implementation, and evaluation of future evidence-based wearable robotic exoskeleton–assisted walking programs offered in the community, and to initiate a reflection regarding the use of wearable robotic exoskeletons during initial rehabilitation following a spinal cord injury.

**Trial Registration:**

ClinicalTrials.gov NCT03989752; https://clinicaltrials.gov/ct2/show/NCT03989752

**International Registered Report Identifier (IRRID):**

DERR1-10.2196/19251

## Introduction

### Deleterious Effects of Nonactive Sitting Time and Reduced Physical Activity

Approximately 100,000 Canadians are currently living with a *spinal cord injury* (SCI) and nearly 4000 new cases are reported annually in Canada [1]. Individuals affected by an SCI usually experience sensory, motor, and autonomic impairments that challenge their walking and walking-related abilities. Despite intensive initial rehabilitation, regaining effective walking ability is challenging for most [2,3]. Indeed, many individuals will not regain their ability to walk due to trunk and lower extremity paralysis or severe paresis. For others, the cardiorespiratory, muscular, or balance requirements needed to walk are too great to achieve a sufficient distance (~183-677 m) or velocity (~0.44-1.32 m/s) for ambulation within their home or in the community [4]. Hence, they will generally use a powered or manually propelled wheelchair as their main mode of mobility. The prolonged, nonactive sitting time [5] and the reduction or cessation of physical activity [6,7] typically linked to this mode of mobility contribute to the development or exacerbation over time of complex and chronic secondary health problems. These health problems often effect the cardiorespiratory [8-12], musculoskeletal [13-17], and endocrine-metabolic [18-23] systems. Moreover, they are often coupled with raised risks of nociceptive or neuropathic pain [24,25] or psychological morbidity [26] (eg, increased depressive symptoms). In turn, these negatively affect functional skills and capacity as well as psychosocial factors in long-term *wheelchair users with a chronic SCI* (WU_SCI_) [27], while also increasing the risk of premature mortality and the burden on caregivers. It is no surprise that these impacts come with substantial financial costs, estimated to be between CAD $1.5 million and $3 million (~US $1.1-$2.3 million) per person with an SCI in the Canadian health care system [28].

### Inventory of Rehabilitation and Physical Activity Interventions

Given the increased life expectancy owing to improvements in medical treatment, along with the growing population of individuals with SCI, there have been recent calls to direct additional attention to the cascade of cardiorespiratory, musculoskeletal, and endocrine-metabolic health problems faced by this population and to interventions targeting modifiable factors linked to these problems [29-31]. To date, most rehabilitation and physical activity interventional studies aiming to mitigate these health problems can be grouped within three main categories: (1) static standing activities using frames with or without full-body vibrations [32], (2) dynamic standing activities combining braces, body-weight support, functional electrical stimulation, or robotic exoskeleton systems for treadmill ambulation with various degrees of lower extremity weight bearing [33-36], and (3) lower extremity or trunk neuromuscular electrical stimulation for cycling or rowing in a sitting position [37,38]. Scoping and systematic reviews confirm that most of these interventions were tested among relatively small and heterogeneous samples of individuals with SCI over relatively short periods of time (ie, ≤12 weeks) [30]. They also highlight that superiority, equivalence, and noninferiority trial designs have rarely been used and no clear consensus has yet emerged on the best possible interventions for a given individual at a specific time within the continuum of care [30]. Nonetheless, based on the currently available evidence, it is relatively well established among WU_SCI_ that the following is true:

1. Gravity-derived high-standing loads, as well as impacts resulting even from low walking speeds [39], are the prominent sources of adaptive stimuli for bone health and surpass the effects linked to static standing or resistance training alone (eg, functional electrical stimulation) [40].
2. Two to three sessions per week of regular structured exercise at moderate-to-vigorous intensity for at least 20 minutes, plus upper body strength exercise (ie, three sets of 10 repetitions at 50%-80% of the one-repetition maximum for large muscle groups), improve cardiorespiratory and endocrine-metabolic health [41-43]. Increasing this exercise intensity is expected to potentiate these beneficial effects [44].

### Wearable Robotic Exoskeletons as a Promising Intervention

**Figure 1:**
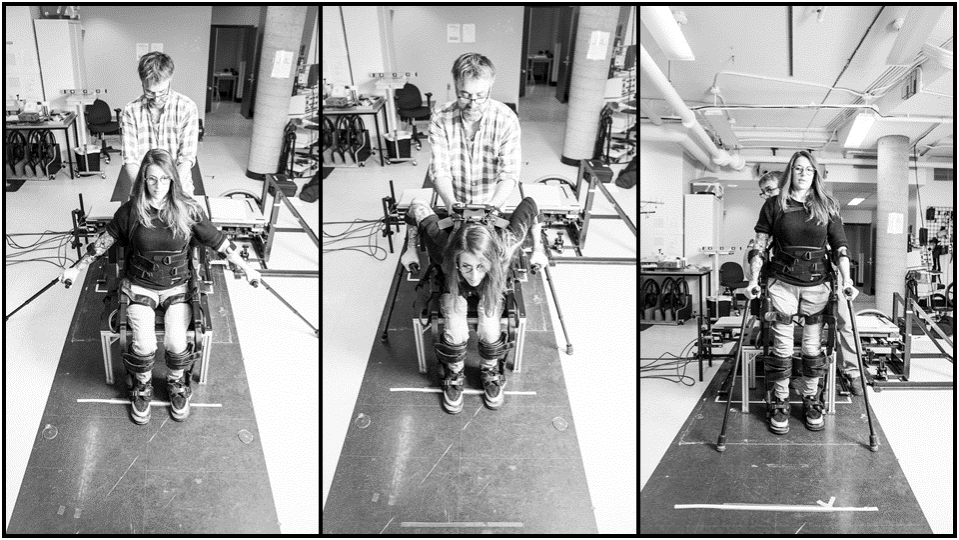
Wearable robotic exoskeleton for sit-stand transitions and overground walking manufactured by Ekso Bionics.

Commercially available wearable robotic exoskeletons, an assistive technology allowing WU_SCI_ to stand and walk overground (see Figure 1), now offer opportunities for clinicians and scientists to develop novel activity-based physical activity programs articulated around overground walking and walking-related abilities [45,46]. Though only small-scale studies are currently available, including a feasibility study from our lab, with most focusing on walking performance [45-54], emerging evidence suggests that performing sit-stand transfers, standing, and walking with a wearable robotic exoskeleton promote lower extremity mechanical loading and mobility. The gravity exposure and muscle elongation-relaxation cycles at the lower extremities signal the mechanosensitive osteocytes and regulate the bone remodeling process via bone formation and reabsorption (ie, mechanotransduction process) [55]. Additionally, the performance of these functional abilities with a wearable robotic exoskeleton solicits the large trunk, thoracohumeral, and upper extremity muscles [56] (ie, *strengthening exercise*) via three mechanisms: (1) maintenance of standing balance, which is even more difficult as the center of mass is moved upward and backward given the configurations of the wearable robotic exoskeleton, (2) control of anterolateral body-weight shifts required to safely initiate the steps, and (3) unloading lower extremities to smooth heel contact with the ground at heel strike. Walking with a wearable robotic exoskeleton also increases the energy expenditure need (ie, *aerobic exercise*) [57-59]. By coupling these types of exercises, walking with a wearable robotic exoskeleton may lead to beneficial cardiorespiratory, musculoskeletal, and endocrine-metabolic [60,61] adaptations in WU_SCI_. In addition, a growing body of evidence suggests that cognitive and executive [62] as well as psychological [63,64] benefits can be anticipated, as favorable associations with physical activity, especially aerobic exercise, have been documented. Also of interest, WU_SCI_ have expressed high levels of satisfaction with wearable robotic exoskeleton–assisted walking programs and have positively perceived the wearable robotic exoskeleton learnability and usability [64]. To what extent these health benefits may have positive synergistic effects on their functional capacities and psychosocial well-being is unclear. How to best configure wearable robotic exoskeleton–assisted walking programs (eg, number, frequency, duration, and intensity of sessions) while conciliating them with the perspectives of WU_SCI_ to potentiate the outcomes of such programs also remains elusive.

### Objectives

The *overall goals* of this study, using mixed methods, are as follows: (1) to determine the immediate and short-term effects of a 16-week overground wearable robotic exoskeleton–assisted walking program on organic systems, functional capacities, and multifaceted psychosocial factors among WU_SCI_ living in the community, and (2) to determine self-reported satisfaction and perspectives with regard to the intervention and the device. The *specific objectives* are articulated around four research questions and hypotheses:

1. Question 1: Does a 16-week walking program with the wearable robotic exoskeleton induce beneficial changes on musculoskeletal, cardiorespiratory, and endocrine-metabolic health; wheelchair-related functional skills and mobility; and psychosocial outcomes? Hypothesis 1: It is hypothesized that beneficial effects observed during the postintervention and retention measurement times will significantly and meaningfully exceed any changes observed during the control and preintervention measurement times (ie, T_0_ [control measurement time] vs T_1_ [preintervention measurement time] vs T_2_ [postintervention measurement time] vs T_3_ [retention measurement time]).
2. Question 2: What personal factors best determine and predict the beneficial effects of the walking program with the wearable robotic exoskeleton? Hypothesis 2: It is hypothesized that the individuals with the highest level of SCI and the longest time since the SCI (ie, possibly the best determinants and predictors) will be those who respond best to the walking program.
3. Question 3: What program attributes best determine and predict the beneficial effects of the walking program with the wearable robotic exoskeleton? Hypothesis 3: It is hypothesized that the total number of steps taken will be the best determinant and predictor of the measured changes.
4. Question 4: What are the participants’ satisfaction levels with the walking program and the wearable robotic exoskeleton itself, and what are the expectations regarding its future use in the context of a home- or community-based adapted physical activity program? Hypothesis 4: It is hypothesized that WU_SCI_ will (1) express high levels of satisfaction with the walking program using the wearable robotic exoskeleton and with the wearable robotic exoskeleton itself and (2) report on how they envision its future in the context of home- or community-based use to shape the development of an adapted physical activity program in the future.

## Methods

### Study Design

**Figure 2:**
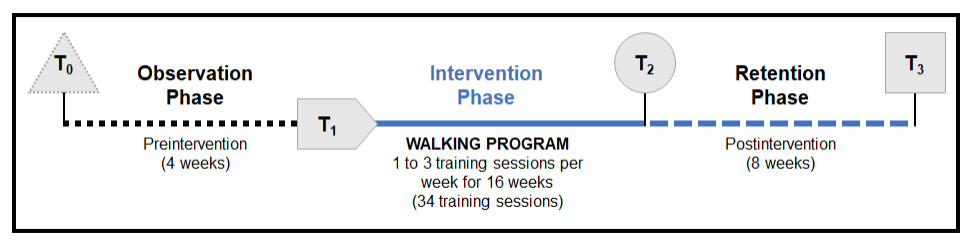
Summary of the design of the study along with the different assessment times. T_0_: control measurement time; T_1_: preintervention measurement time; T_2_: postintervention measurement time; T_3_: retention measurement time.

A prospective, longitudinal, self-controlled interventional study with multiple discrete measurement times will be used to assess outcomes at baseline (ie, preintervention phase), during the intervention, and thereafter (ie, retention phases) (see Figure 2). While this design may contrast with *classic* study designs frequently recommending a separate comparison group to assess efficacy or effectiveness, the use of a separate comparison group was judged as nonideal in the context of this study, based on the challenges linked to the classification and quantification of the severity of SCIs and their heterogeneous consequences [65]. In addition, interventional trials of new technologies document that potential participants are reluctant to participate, refuse to adhere to the requirements of a control group, or withdraw for the most part from a control group [66,67]. To strike an optimal balance between the need to have a control group with the anticipated strong desire of potential participants to engage in the wearable robotic exoskeleton–assisted walking program, while also considering the amount of, and timeline limits linked to, the study’s funding, the study includes a 4-week observation phase prior to the start of the intervention. Since only individuals with a chronic SCI with *stable* overall health status and life habits will be recruited, outcome measures will be assessed at the start and at the end of the 4-week observation phase. These outcome measures will provide data about each participant’s natural variability and will enable us to detect whether the intervention has an effect greater than the underlying natural variability (ie, patient-specific minimal detectable change criteria computed); they will also enable us to test the effects of the intervention (ie, pre- vs postintervention responses). This boosts the ethical acceptability of the project, minimizes the impacts of potential and unmeasured confounding variables, facilitates recruitment, and mitigates the risk of experimental attrition. Lastly, semistructured interviews and an online questionnaire will capture participants’ satisfaction levels and perspectives about the wearable robotic exoskeleton–assisted walking program and the mobility technology in itself (ie, the wearable robotic exoskeleton).

### Participants and Inclusion and Exclusion Criteria

We aim to recruit a nonprobabilistic consecutive sample of 20 long-term WU_SCI_. The inclusion and exclusion criteria are listed in Textbox 1. Initial screening is conducted by phone to establish eligibility based on criteria developed during the feasibility study. Once deemed eligible from the initial clinical screening, potential participants attend a short assessment to confirm eligibility.

**Inclusion and exclusion criteria.**

Participant-specific inclusion criteria:

- Adults (≥18 years old)
- Chronic complete or incomplete traumatic or nontraumatic spinal cord injury (SCI) at least 18 months before enrollment
- Long-term manual wheelchair use as primary means for in-house and community mobility (ie, nonambulatory)
- Understand and communicate in English or French
- Reside or will arrange for temporary housing in the community within 75 km from the main research site

Participant-specific exclusion criteria:

- Other neurological impairments aside from those linked to the SCI (eg, multiple sclerosis)
- Concomitant or secondary musculoskeletal impairments (eg, hip heterotopic ossification)
- History of lower extremity fracture within the past year
- Unstable cardiovascular or autonomic system
- Renal insufficiency
- Pregnancy
- Any other conditions that may preclude lower extremity weight-bearing, walking, or exercise tolerance in the wearable robotic exoskeleton

Exoskeleton-specific inclusion criteria:

- Body mass: ≤100 kg
- Height: 1.52-1.93 m
- Pelvis width: 30-46 cm
- Thigh length: 51.0-61.4 cm
- Lower leg length: 48.0-63.4 cm

Exoskeleton-specific exclusion criteria:

- Inability to sit with hips and knees at ≥90° flexion
- Lower extremity passive range of motion limitations (hip flexion contracture ≥5°, knee flexion contracture ≥10°, and ankle dorsiflexion ≤–5° with knee fully extended)
- Moderate-to-severe lower extremity spasticity (score of >3 on the Modified Ashworth Scale)
- Length discrepancy (≥1.3 cm or ≥1.9 cm at the thigh or lower leg segment, respectively)
- Skin integrity issues preventing wear of the wearable robotic exoskeleton

### Intervention: Overground Wearable Robotic Exoskeleton–Assisted Walking Program

**Figure 3:**
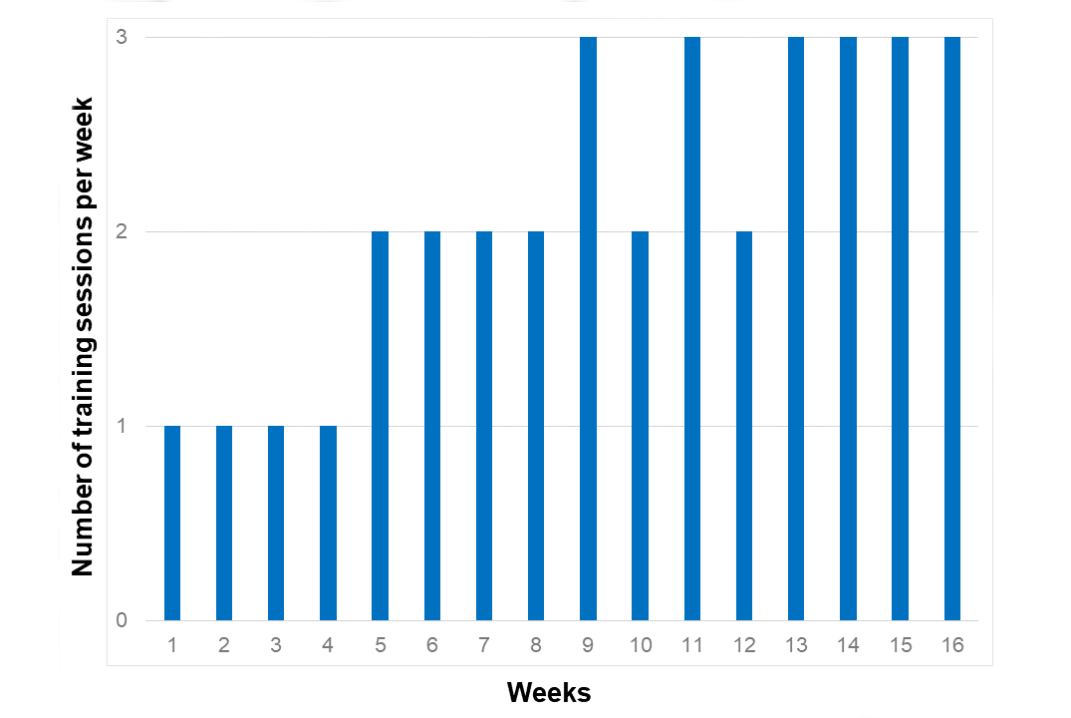
Progression of the number of training sessions per week during the 16-week walking program.

An Ekso GT (Ekso Bionics) wearable robotic exoskeleton, which has been approved by Health Canada, is used in this study (see Figure 1). At T_1_, participants engage in a wearable robotic exoskeleton–assisted overground walking program that encompasses 34 training sessions offered over a 16-week period. The number of training sessions per week progressively increases to safely and efficiently adhere to the overload principle (see Figure 3). The duration of the training program mimics that of a recent study that confirms for the first time an improvement in bone turnover among individuals with a chronic SCI following a 16-week walking program [39]. The frequency of the training program that progresses from one to three sessions per week matches the latest recommendation from the Physical Activity Guidelines for Adults with SCI, including the new conditional recommendation to engage in at least 30 minutes of moderate-to-vigorous intensity aerobic exercise three times per week for cardiometabolic health benefits [68]. All training sessions are supervised by a certified physiotherapist as well as a physiotherapy assistant as needed [46,53]. During each 60-minute training session, participants perform sit-stand transfers and walk with the wearable robotic exoskeleton and a walking aid (ie, rolling walker or forearm crutches). Verbal and tactile feedback are provided by the physiotherapist as needed.

Total hip areal bone mineral density (aBMD), determined with dual-energy x-ray absorptiometry (DXA) scans performed at T_1_, is used to assign each participant to one of three training regimes based on lower extremity fracture risks [69]: (1) conservative (T-score ≤ –2.5; first session includes a maximum of 300 steps; number of steps progresses up to 10% every week), (2) moderate (–2.5 < T-score < –1.0; first session includes a maximum of 400 steps; number of steps progresses up to 15% every week), or (3) aggressive (T-score ≥ –1.0; first session includes a maximum of 500 steps; number of steps progresses up to 20% every week). Workload is further individualized depending on each participant’s level of proficiency and tolerance; it is progressively and safely increased by modifying walking parameters (eg, number of steps, speed, and duration) or reducing total resting time or level of assistance provided by the physiotherapist to maintain a moderate-to-vigorous training intensity (ie, rate of perceived exertion ≥3/10 on the Modified Borg Scale [70]). Training parameters are recorded at the end of each session (eg, total standing time, total walking time, total number of steps, assistance provided, and rate of perceived exertion). Given the risks of adverse events inherently linked to the use of a wearable robotic exoskeleton [69,71], skin integrity at interface pressure points, particularly at the tibial tuberosity, and signs of inflammation at the ankle and knee joints before and after each training session, respectively, are assessed and any serious adverse events will be reported.

To be considered as having successfully completed the program, at least 75% of the training sessions (ie, 26/34) need to have been completed. To this effect, to assure an optimal attendance rate similar to the one reached during the feasibility study (ie, attendance rate was 97.7%) [72] and to overcome one of the most commonly reported barriers to intervention studies (ie, transportation), participants have three key options: (1) driving their own car or being transported by car by a family member or a friend with free parking provided within 50 meters from the entrance, (2) using public transit, or (3) scheduling trips with the adapted transport free services.

### Outcomes: Domains, Tools, Measures, and Assessment Times

#### Overview

**Figure 4:**
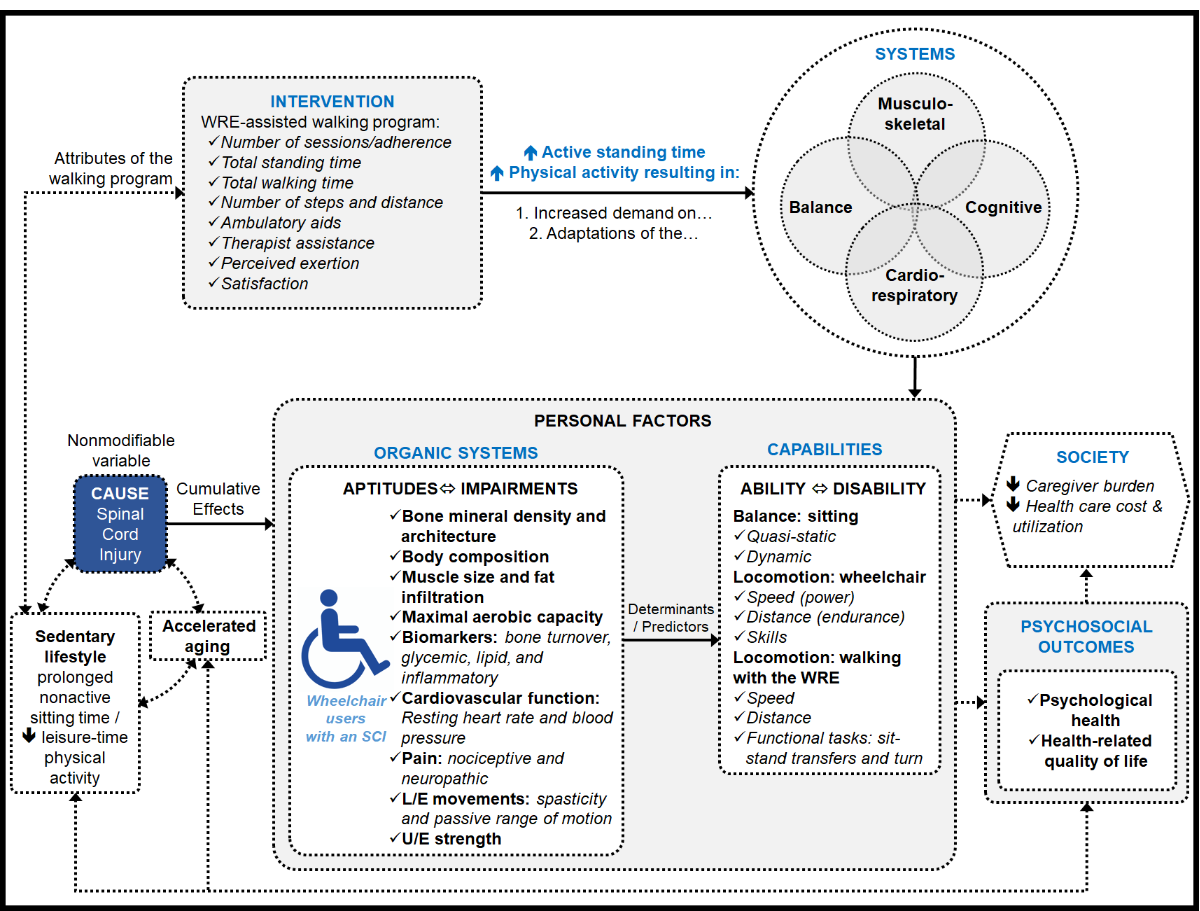
Project-specific logic model highlighting the relationships between the different domains of interest and related outcome measures. L/E: lower extremity; SCI: spinal cord injury; U/E: upper extremity; WRE: wearable robotic exoskeleton.

All outcomes reflecting the potential impacts of the intervention based on the logic model (see Figure 4) are prospectively collected at T_0_, T_1_, T_2_, and T_3_, except the participants’ satisfaction levels and perspectives and the endpoint interviews, which are only completed at T_2_. Outcomes are collected by a registered physiotherapist (AB) who has been trained with standardized data collection protocols adapted for WU_SCI_. All selected outcomes are commonly used in clinical trials targeting similar domains and populations—most have been used in the feasibility study—and most are summarized, including psychometric properties, and are recommended by well-established medical, rehabilitation, or psychosocial research organizations and networks [73]. A summary of all outcomes included in this study and the times at which they are administered is provided in Table 1. The following subsections describe all outcomes in detail.

**Table 1:** Summary of outcomes.

Outcomes			Measurement times^a^			
			T_0_	T_1_	T_2_	T_3_
**Clinical assessments**						
	**Personal characteristics**					
		Sociodemographic characteristics (age, sex, etc)	✓			
		Neurological impairment (American Spinal Injury Association Impairment Scale)	✓			
		Anthropometric parameters (weight and height)	✓			
		Resting heart rate and blood pressure	✓	✓	✓	✓
		Pain (International SCI [spinal cord injury] Pain Basic Dataset version 2.0)	✓	✓	✓	✓
		Passive range of motion at the ankle, knee, and hip joints (two-axis goniometer)	✓	✓	✓	✓
		Spasticity (Modified Ashworth Scale)	✓	✓	✓	✓
	**Wheelchair abilities**					
		20-meter wheelchair propulsion test (natural and maximal speeds)	✓	✓	✓	✓
		Slalom test	✓	✓	✓	✓
		6-minute manual wheelchair propulsion test	✓	✓	✓	✓
**Laboratory assessments**						
	**Bone mineral density and architecture**					
		Dual-energy x-ray absorptiometry (hip and lumbar vertebrae)	✓	✓	✓	✓
		Peripheral quantitative computed tomography (proximal tibia, distal femur, and proximal radius)	✓	✓	✓	✓
	**Body composition**					
		Dual-energy x-ray absorptiometry (total body)	✓	✓	✓	✓
	**Muscle quality**					
		Peripheral quantitative computed tomography (intramuscular fat infiltration)	✓	✓	✓	✓
	**Blood biomarkers**					
		Bone turnover (serum procollagen type 1 N-terminal peptide, osteocalcin, C-terminal cross-linking telopeptide, and 25-hydroxyvitamin D)	✓	✓	✓	✓
		Glycemia (fasting glucose, insulin, and glycosylated hemoglobin)	✓	✓	✓	✓
		Insulin resistance (homeostatic model assessment)	✓	✓	✓	✓
		Lipids (total cholesterol, high-density lipoprotein, low-density lipoprotein, triglycerides, and apolipoprotein B)	✓	✓	✓	✓
		Inflammation (C-reactive protein, tumor necrosis factor alpha, interleuken-6, and interleuken-10)	✓	✓	✓	✓
	**Cardiorespiratory fitness**					
		Respiratory gas analysis during 6-minute manual wheelchair propulsion test		✓	✓	
		Total distance travelled during the 6-minute manual wheelchair propulsion test	✓	✓	✓	✓
**Take-home assessments**						
	**Psychological health**					
		World Health Organization Quality of Life assessment	✓	✓	✓	✓
		Beck Depression Inventory	✓	✓	✓	✓
		Beck Anxiety Inventory	✓	✓	✓	✓
		Psychological General Well-Being Index	✓	✓	✓	✓
	**Participant satisfaction and perspectives**					
		Updated version of the Montreal Walking Exoskeleton Satisfaction and Perspectives Questionnaire			✓	
		Semistructured interview			✓	

^a^Measurement times: T_0_ (control measurement time), T_1_ (preintervention measurement time), T_2_ (postintervention measurement time), and T_3_ (retention measurement time).

#### Clinical Assessments

##### Personal Characteristics

Assessments are completed at the different assessment times to collect outcomes characterizing the following:

1. Sociodemographic characteristics (eg, age; sex; time since injury; history of fragility fracture; medications, including opioid analgesia, benzodiazepines, or unfractionated heparin; current smoking status; and alcohol intake) [74].
2. Neurological impairment (eg, American Spinal Injury Association Impairment Scale for neurological level, motor and sensory scores, and severity) [75].
3. Anthropometric parameters (eg, weight and height).
4. Resting heart rate and systolic and diastolic blood pressure using an electronic sphygmomanometer machine [76].
5. Pain using the *International SCI Pain Basic Dataset version 2.0*, which includes a set of core questions for up to three separate pain problems experienced over the past week and three questions on perceived pain interference with activities, mood, and sleep [77].
6. Passive range of motion at the ankle, knee, and hip joints with a two-axis goniometer (ie, contracture).
7. Upper extremity muscle strength (ie, pushing and pulling strength with a wheelchair wheel attached to an instrumented dynamometer and handgrip strength with a handheld dynamometer).
8. Lower extremity spasticity using the *Modified Ashworth Scale* [78].

##### Wheelchair Abilities

Wheelchair abilities are assessed using the following performance-based wheelchair propulsion tests: (1) 20-meter wheelchair propulsion test (natural and maximal speeds), (2) slalom test, and (3) 6-minute manual wheelchair propulsion test [79-82]. The slalom test is used as a surrogate of trunk control since forward reaching distance is a good determinant (*r*=–0.75) [80] and trunk control is known to be the best predictor of multidirectional seated limits of stability during reaching (*R^2^*=0.95) [83]. The *Wheelchair Skills Test Questionnaire version 5.0* [84] is used as well to assess wheelchair abilities. This questionnaire assesses 34 wheelchair mobility and wheelchair-related skills. Each skill is rated on a 4-point self-reported scale: responses to the question *Can you do it?* include 0 (no), 1 (partially), 2 (yes), and 3 (very well). A capacity score is calculated (0%-100%), reflecting the number of skills that can be partially or completely done. Moreover, it assesses how confident—*How confident are you?* 0 (not at all), 1 (partly), 2 (moderately), or 3 (very)—and how often—*How often do you do it?* 0 (never), 1 (occasionally), 2 (usually), or 3 (always)—each skill is performed to calculate confidence and performance scores (0%-100%).

#### Laboratory Assessments

##### Bone Mineral Density and Architecture

A DXA system (Lunar Prodigy, GE Healthcare) is used to calculate aBMD at the hip, femoral neck, and the first to the fourth lumbar vertebrae [85]. Moreover, T-scores compare the measured aBMD values of each participant against values predicted from a matched reference group for sex, age, and ethnicity and are expressed as the number of SDs (ie, z scores or T-scores depending on age of participant). Measurements of aBMD and z scores or T-scores are directly provided by the system's software.

In addition, a peripheral quantitative computed tomography (pQCT) system (XCT 3000, Stratec Biomedical Systems) is used to characterize the volumetric bone mineral density (vBMD) and the microarchitecture parameters of trabecular and cortical bones at various imaging sites: 66% of the tibia, 25% of the femur, and 66% of the radius. These sites were chosen to maximize muscle circumference in each scan [86]. To minimize site-selection error between measurement times, images are taken using a standardized protocol including scout views with recommended reference lines [86]. As per current recommendations, reported pQCT outcomes will minimally include the following: total trabecular and cortical mineral content; cortical cross-sectional area and thickness; and biomechanical strength indices calculated from density and area (ie, bone strength indices, polar section modulus, and polar strength strain index) [86]. Outcomes are calculated using a validated open source image analysis software package (Fiji distribution of ImageJ) [87,88].

##### Body Composition

Whole-body scans obtained with the DXA system are used to quantify total and regional (ie*,* upper extremities, trunk, and lower extremities) body fat and fat-free (ie, lean) tissue mass and relative percentages, respectively [89]. These measures are directly provided by the DXA system's software.

##### Muscle Quality

Cross-sectional images of the femur, tibia, and radius captured with the pQCT system are also used to measure the muscle size (ie, cross-sectional area) and intramuscular fat infiltration (ie, muscle density) using the same validated open source image analysis software [87,88].

##### Blood Biomarkers

Fasting blood samples (ie, >8-hour fast) are used to quantify bone turnover biomarkers (ie, serum procollagen type 1 N-terminal peptide, osteocalcin, C-terminal cross-linking telopeptide, and 25-hydroxyvitamin D), glycemic biomarkers (ie, fasting glucose, insulin, and glycosylated hemoglobin), insulin resistance biomarkers (ie, homeostatic model assessment), lipid biomarkers (ie, total cholesterol, high-density lipoprotein, low-density lipoprotein, triglycerides, and apolipoprotein B), and inflammatory biomarkers (ie, C-reactive protein, tumor necrosis factor alpha, interleuken-6, and interleuken-10).

##### Cardiorespiratory Fitness

At T_1_ and T_2_, participants complete the 6-minute manual wheelchair propulsion test wearing a gas analyzer system (COSMED K4b2, COSMED srl). This portable system incorporates a sealed face mask placed over the mouth and nose and anchored around the head, a telemetric stationary O_2_ and CO_2_ gas analyzing unit, and a battery harnessed to the anterior and posterior thorax. This system is calibrated before each test as recommended by the manufacturer. For the other two measurement times (ie, T_0_ and T_3_), the total distance travelled during the 6-minute manual wheelchair propulsion test is used as a surrogate measure of cardiorespiratory fitness since it has been found to strongly correlate and agree with the maximal arm-crank test (*r*=0.92; mean difference 0.21 ±1.94 mL/kg·min) [90].

#### Take-Home Assessments

##### Psychological Health

For the psychosocial outcomes, health-related quality of life is measured with the short version of the *World Health Organization Quality of Life* assessment [91-94]. This includes 24 questions organized around four domains: physical health (seven items), psychological health (six items), social relationships (three items), and environment (eight items). There are two additional questions on overall health-related quality of life and general health. Each question is rated on a 5-point Likert interval scale ranging from 1 (poor) to 5 (good). At the end, scores for each question and a mean score computed for each domain are reported. To further investigate psychosocial outcomes, the *Beck Depression Inventory* [95], the *Beck Anxiety Inventory* [96], and the *Psychological General Well-Being Index* are used [97-99]. The *Beck Depression Inventory* and the *Beck Anxiety Inventory* are each comprised of 21 groups of statements that are evaluated on 4-point Likert scales, with higher scores indicating higher depressive or anxiety-related symptoms, respectively. The *Psychological General Well-Being Index* includes 22 items organized around components of psychological well-being, such as anxiety, positive well-being, self-control, depression, and general health and vitality. Response options for each item are individualized according to the given affective experience. Intensity or frequency of experience during the past month is rated on a 6-point Likert scale ranging from 0 (most negative option) to 5 (most positive option).

##### Participant Satisfaction and Perspectives

For participant satisfaction, an updated version of the *Montreal Walking Exoskeleton Satisfaction and Perspectives Questionnaire* (MWESP-Q) is completed online at T_2_. This questionnaire includes 54 statements that are organized around seven key domains: overall satisfaction related to the training program (two statements); satisfaction related to the overground robotic exoskeleton (seven statements); perceived learnability (12 statements); satisfaction related to the program attributes (eight statements); perceived health benefits (12 statements), including sentences that relate to pain, spasticity, bowel functions, and sleep; perceived risks and fears (11 statements); and perceived motivation to engage in regular physical activity (two statements). Each statement is rated using a 7-point Likert scale ranging from 1 (most negative option; eg, strongly disagree) to 7 (most positive option; eg, strongly agree) [64].

For participants’ perspectives, a 20-30-minute semistructured interview is conducted over the phone to capture their general experience when participating in the wearable robotic exoskeleton–assisted training program. The interviews also serve as a platform for documenting participants’ perspectives on the future of wearable robotic exoskeleton technology. To do so, various themes are discussed: potential benefits and recommendations for wearable robotic exoskeleton–assisted walking during the acute and subacute phases following spinal cord injury (eg, timing and conditions) or in a clinical setting during the chronic phase; opportunities for improvement (eg, functionality and structural aspects of the wearable robotic exoskeleton); and recommendations for a future home- or community-based wearable robotic exoskeleton–assisted walking program (eg, stairs, different surfaces, donning and doffing the wearable robotic exoskeleton without assistance, and operating the wearable robotic exoskeleton without assistance). The research professional who conducts these interviews has never met the participants and is not a member of the research team. All interviews are recorded to later enable verbatim transcription.

### Statistics

#### Sample Size Estimation

The sample size estimate was based on a comparison using the variability of the absolute change (ie, mean ±SD) in both body composition and bone mineral density (ie, main outcomes) measured pre- and postintervention in our preliminary study [61] and computed with a computerized sample size calculator [100]. Considering the self-controlled design of the study, a total of 18 participants are required to have an 80% chance of detecting a significant increase at the 5% level in the leg lean body mass (kg) at the tibia measured by DXA from 14.0 to 15.8 ±2 mg/cm^3^ [61]. Likewise, a total of 18 participants are required to have an 80% chance of detecting a significant increase at the 5% level in the bone mineral density (mg/cm^3^) at the tibia measured by pQCT from 466 to 532 ±70 mg/cm^3^ [61]. Considering the dropout rate of 7.1% found during the feasibility study [46], an additional 2 participants were added for a total sample size of 20 participants.

#### Quantitative and Qualitative Analyses

Descriptive statistics (eg, mean, SD, and 95% confidence interval) will be calculated for data summarizing sociodemographic characteristics as well as clinical and laboratory outcomes collected at the different measurement times. The normality of all data distributions and the absence of outliers will be verified via the Shapiro-Wilk test of normality and the absence of studentized residuals greater than ±3 SDs, respectively. Whenever applicable, the level of significance will be set at *P*≤.05 for all statistical tests and the data will be analyzed using SPSS, version 25.0 (IBM Corp).

For Hypothesis 1, one-way analysis of variance for repeated measures (ie, normally distributed continuous data) or Freidman tests (ie, non-normally distributed continuous or categorical data) with planned comparisons based on the hypothesis and Bonferroni correction will be conducted to detect significant time effects, with a special interest for the preintervention (T_0_ vs T_1_), intervention (T_1_ vs T_2_), and retention (T_2_ vs T_3_) phases. In accordance with the principles of a classic intention-to-treat approach, all participants will be included in the final analyses, regardless of withdrawal, compliance, or unintentional missing data. For missing data, imputation of the mean value for the specific group at the specific assessment time will be used. Per-protocol exploratory analyses will also be performed comparing outcomes for those with walking program compliances of greater than 75% to examine maximum treatment efficacy.

For Hypotheses 2 and 3, Pearson or Spearman correlation coefficients will investigate the strength and direction of the relationships between the overall observed changes ([T_1_–T_2_] / T_1_ × 100) for each personal factor (ie, dependent variable) and the program characteristics (ie, independent variable). Independent variables having reached a critical threshold (*P*≤.25) will then be confronted in a stepwise multiple linear regression analysis to identify the three best predictors for each dependent variable and the coefficient of determination (*R^2^*) of the model. To determine the factors for identifying the best responders to the program, analyses similar to the previous ones will be completed, except that the independent variables will be sociodemographic characteristics, anthropometric parameters, characteristics of the SCI, personal factors, and functional disability.

For Hypothesis 4*,* using Microsoft Excel, descriptive statistics of the 54 statements (ie, mean, median, and SD) will summarize the results of the satisfaction survey (ie, the MWESP-Q). Audio recordings of the interviews will be transcribed verbatim using Microsoft Word. A summer research intern will read the transcripts and generate initial codes or subthemes. A co-codification of the first transcript will be done manually on printed paper with the list of initial codes and subthemes; this will be done independently by three research collaborators (ie, a summer research intern, a doctoral student, and a researcher who is a member of the research team). They will write codes directly in the margin of the printed transcripts. They will then meet via audio and video conferencing to compare and review coding, discuss discrepancies, and modify codes if necessary. This process will be done twice with the transcripts of the first two participants. To start the computerized qualitative analysis, all transcripts will be interpreted using thematic content analysis, where narrative data will be thematically coded and appraised using NVivo 10 (QSR International). A final report, integrating findings from the MWESP-Q and the interviews, will be produced and recommendations will be incorporated in order to shape the development of a future home- or community-based adapted physical activity program.

## Results

This study was recently initiated at the *Laboratoire de pathokinésiologie* of the Centre de recherche interdisciplinaire en réadaptation du Montréal métropolitain (CRIR), Québec, Canada, which is part of the Centre intégré universitaire de santé et de services sociaux du Centre-Sud-de-l’Ile-de-Montréal in Montreal, Canada, and at the *Laboratoire du muscle et de sa fonction* of the Université du Québec à Montréal. This project received ethical approval on March 14, 2019, from the CRIR ethics committee and was registered on June 7, 2019, with the US National Library of Medicine at ClinicalTrials.gov (NCT03989752). This study is expected to be completed by spring 2021, with results to follow shortly after.

## Discussion

### Overview

This project innovates by being among the first studies to comprehensively, prospectively, and longitudinally investigate the effects of a wearable robotic exoskeleton–assisted walking program among long-term WU_SCI_ who have a very poor prognosis for walking recovery [101]. This study investigates the effects on organic systems, functional capacities, and multifaceted psychosocial factors. This study also investigates the influence of walking program attributes (eg, duration, training frequency, and intensity) on these effects. This project is crucial in strengthening evidence in this field based on hierarchical forms of knowledge creation. This project is also timely, since WU_SCI_ are now requesting the democratization of accessibility to this technology during rehabilitation and in the community at a faster rate than evidence is generated and shared with physical activity and rehabilitation professionals, administrators, and policy makers. Strengthened evidence is urgently needed to fill this knowledge gap to some extent and to start informing the decision-making process of these stakeholders regarding the possibility of purchasing wearable robotic exoskeletons as well as developing, implementing, and evaluating activity-based and adapted physical activity programs with wearable robotic exoskeletons in clinical practice (eg, walking program). Moreover, this evidence, once coupled with clients’ perspectives, may become key precursors to (1) develop and implement community- and/or home-based walking programs, (2) advocate for policy changes to broaden accessibility to wearable robotic exoskeletons, and (3) propel future larger-scale pragmatic or randomized controlled trials with an appropriate comparator targeting the effectiveness of walking programs with a wearable robotic exoskeleton. Of even greater relevance, it is expected that walking programs with a wearable robotic exoskeleton will reduce impairments (ie, organic systems) and optimize aptitudes, which are expected to positively influence psychosocial outcomes in WU_SCI_ and potentially those with other sensorimotor impairments (eg, stroke). In the long term, although not measured in the proposed study, we can hypothesize that indirect societal benefits may become tangible via reduced caregiver burden and reduced health care costs, for example. By optimizing their global health, especially as they age, WU_SCI_ will also remain in a *state of readiness* to benefit from future advances in neural repair, recovery, or rehabilitation technology. Lastly, as some wearable robotic exoskeletons may become approved for home or community use by Health Canada, some WU_SCI_ are now envisioning their use as personal neuroprostheses for ambulation in their daily lives as a complement to wheelchair mobility in the near future. Still, strengthened evidence is needed to support the prescription and reimbursement processes in order to accelerate uptake of wearable robotic exoskeletons in the community.

Many stakeholders may benefit from this interventional study. For WU_SCI_ who have no or very limited walking ability, the walking program with the wearable robotic exoskeleton is not expected to have any reversal effect on their walking capacity without this novel mobility assistive technology. However, this project is relevant since it will generate the first evidence of the anticipated cardiorespiratory, musculoskeletal, and endocrine-metabolic health adaptations upon completion of a walking program with a wearable robotic exoskeleton. For the first time, the extent to which these adaptations translate into beneficial effects on functional capacity will also be verified, as will their effects on health-related quality of life and psychological health. This includes the psychological well-being domain, which was not specifically measured during the feasibility study but was mentioned by the majority of participants. Given the fact that the population of WU_SCI_ continues to grow and that they now live longer, these potential beneficial effects are further warranted. The caregiver burden and the potentially costly long-term expenditures associated with adverse health events may also decrease. For rehabilitation professionals, the proposed project is relevant since strengthened evidence regarding the effects of the walking program and the characteristics of the best responders will be generated and will inform clinical decision-making processes or the development of a clinical algorithm for referring individuals with SCIs to a walking program. For rehabilitation program administrators and policy makers, the proposed study is relevant since the evidence generated may further confirm the need for publicly funded clinical and technological infrastructures to create structured programs incorporating walking technologies, such as the wearable robotic exoskeleton, and outcome measures into rehabilitation or adapted physical activity centers. Both program administrators and policy makers will need to work collaboratively and cohesively to develop creative solutions to address this current service gap and engage in transformative improvements. For the research community, this project provides a unique opportunity to create a strong multidisciplinary team of well-established scientists with diverse and complementary academic training as well as clinical and fundamental research expertise. For manufacturers with an interest in wearable robotic exoskeletons, among others, this project is relevant since the input from powered exoskeleton end users (ie, WU_SCI_) will become available and may enrich the continuous quality improvement process. This process is imperative to further support and accelerate the development of wearable robotic exoskeletons and to reach key commercialization milestones for the technology to become personalized and accessible for WU_SCI_ interested in home or community use (ie, neuroprosthesis) in the next decade.

### Potential Challenges and Appropriate Mitigation Strategies

A few potential challenges merit attention:

1. Some potential participants will have insufficient passive range of motion at the lower extremities to engage with the project. These participants will be provided with a 4- to 6-week home-based stretching program, will be reassessed, and may become eligible later.
2. Female WU_SCI_ may be underrepresented. Efforts will be made for the sample to be representative of the SCI population and to have women make up 20% of the sample. However, since the minority of individuals affected by SCIs are female and the sample size is limited (n=20), it is unlikely that statistical analysis by subgroups (ie, male vs female) will be feasible in order to account for potential sex and gender differences. Nonetheless, descriptive statistics will present results separately for women and men whenever indicated.
3. A small number of participants may demonstrate vitamin D deficiencies [102]. To mitigate this risk, at T_0_, the equivalent of 1 year of vitamin D3 supplementation will be provided to all participants who are not currently taking supplements. Moreover, all participants will be instructed on healthy balanced diets.
4. Some participants may concurrently engage in extraneous physical activity or may seek cointerventions during the project. Participants will be asked to maintain their customary level of physical activity during the project and to avoid engaging in new cointerventions. Any unintended intervention (ie, contamination or cointervention) that may influence the results will be documented and its effect carefully verified by the research team and possibly considered as a dichotomous variable (ie, present vs absent).
5. Some participants may experience some lower extremity neurorecovery. In the event a participant was to experience neurorecovery (ie, lower extremity motor score of ≥20 on the American Spinal Injury Association Impairment Scale), he or she would be withdrawn from the project and referred for a comprehensive neurological assessment and to an advanced locomotor training program.

## Abbreviations

aBMD: areal bone mineral density
CRIR: Centre de recherche interdisciplinaire en réadaptation du Montréal métropolitain
DXA: dual-energy x-ray absorptiometry
MWESP-Q: Montreal Walking Exoskeleton Satisfaction and Perspectives Questionnaire
pQCT: peripheral quantitative computed tomography
SCI: spinal cord injury
T_0_: control measurement time
T_1_: preintervention measurement time
T_2_: postintervention measurement time
T_3_: retention measurement time
vBMD: volumetric bone mineral density
WU_SCI_: wheelchair users with a chronic spinal cord injury
